# Neonatal sclerosing cholangitis with novel mutations in *DCDC2* (doublecortin domain-containing protein 2) in Chinese children

**DOI:** 10.3389/fped.2023.1094895

**Published:** 2023-02-03

**Authors:** Xia Wei, Yuan Fang, Jian-She Wang, Yi-Zhen Wang, Yuan Zhang, Kuerbanjiang Abuduxikuer, Lian Chen

**Affiliations:** ^1^ Department of Pathology, Anhui Provincial Children's Hospital, Hefei, China; ^2^ Department of Hepatology, Children's Hospital of Fudan University, Shanghai, China; ^3^ Department of Ultrasound, Children's Hospital of Fudan University, Shanghai, China; ^4^ Department of Pathology, Children's Hospital of Fudan University, Shanghai, China

**Keywords:** neonatal sclerosing cholangitis, biliary cirrhosis, *DCDC2*, ciliopathy, novel mutations

## Abstract

**Background:**

Neonatal sclerosing cholangitis (NSC) is a rare and severe autosomal recessive inherited liver disease with mutations in *DCDC2*, commonly requiring liver transplantation (LT) for decompensated biliary cirrhosis in childhood.

**Methods:**

The information of four Chinese patients with NSC caused by mutations in *DCDC2* from Children's Hospital of Fudan University were gathered. The four patients' clinicopathological and molecular features were summarized by clinical data, liver biopsy, immunohistochemical, and molecular genetic analysis.

**Results:**

All patients presented with jaundice, hepatosplenomegaly, hyperbilirubinemia and bile embolism, and high serum *γ*-glutamyl transferase activity (GGT). Liver biopsies revealed varying degrees of bile duct hyperplasia, portal-tract inflammation, and/or fibrosis. Whole-exome sequencing (WES) found novel heterozygous variants of c.1024-1G > T /p.? and c.544G > A /p. Gly182Arg in the *DCDC2*.

**Conclusion:**

This study expands the genetic spectrum of *DCDC2* in NSC.

**Author Summary:** Neonatal sclerosing cholangitis (NSC) is an uncommon and severe inherited liver disease, which mutations in *DCDC2* are responsible for, often leading to liver transplantation (LT) for end-stage liver disease in childhood. Herein, we collected the information of four Chinese patients with NSC caused by mutations in *DCDC2* and analysed these patients’ clinical, pathological, and molecular features. We identified novel mutations (c.1024-1G > T and c.544G > A) in *DCDC2*, which have not been reported. This study adds to the genotype spectrum of *DCDC2* causing NSC and expands the understanding of this rare disease.

## Introduction

Neonatal sclerosing cholangitis (NSC, OMIM 617394) is a rare and severe neonatal-onset cholestatic liver disease, which ultimately develops into end-stage liver disease and has to undergo liver transplant in childhood (1). The main clinical manifestations include jaundice, hepatosplenomegaly, hyperbilirubinemia and high serum gamma glutamyl transferase activity (GGT), which was firstly reported in 1987 (2). It is difficult to distinguish from biliary atresia and other neonatal cholestatic liver disease in early NSC (3). Typical imaging and liver biopsy pathology can assist in establishing diagnosis. At the early stage of the disease, the typical cholangiogram features of NSC are as follows: unobstructed bile ducts, thin and irregular intrahepatic duct with or without extrahepatic bile ducts change (4). Liver biopsies often revealed bile duct hyperplasia, portal-tract inflammation, and/or fibrosis. In 2004, Hadj-Rabia et al. firstly reported NSC caused by claudin 1 (*CLDN1*) gene associated with ichthyosis, which encodes a tight-junction protein (5). In 2016, Girard et al. (4)and Grammatikopoulos et al. (6) verified that doublecortin domain containing 2 (*DCDC2*) gene mutations can lead to NSC. Apart from that, *DCDC2* pathogenic variants were reported to be associated with renal-hepatic ciliopathy with nephronophthisis, non-syndromic recessive deafness, dyslexia and central nervous system impairment (7–10). With the development of whole-exome sequencing (WES), more and more *DCDC2* mutations have been identified. To our knowledge, seventeen cases of NSC caused by *DCDC2* mutation with 12 variants have been reported worldwide (4, 6, 10–14).

In this study, we summarized the clinical and laboratory features, presentation, and disease progression of NSC in four Chinese children caused by *DCDC2* mutation, two of whom have been reported in our previous publication (11). The Liver biopsies showed various degrees of bile duct hyperplasia, portal-tract inflammation, and/or fibrosis. WES detected compound heterozygous variants c.1024-1G > T and c.544G > A, and homozygous variant c.529dupA in *DCDC2*, of which the former had not been previously reported. All patients were treated with Ursodeoxycholic acid capsules, Cholestyramine, Vitamins A, D, E and K1 to relieve symptoms and prevent the rapid progression of the disease. Over time, the symptoms of all patients have improved during follow-up. Our study identified novel mutations in *DCDC2* and expanded the molecular spectrum of NSC.

## Materials and methods

### Patient

The inclusion criteria included patients had the disorder of liver function, cholestasis with elevated GGT during infancy, in whom cholangiopathy was demonstrated on histopathology or imaging. Meanwhile, WES confirmed mutations in only *DCDC2*. Moreover, the cholangiography showed no atresia of the bile duct. Then the patient can be definitively diagnosed with a *DCDC2*-related NSC. Exclusion criteria covered biliary atresia, ichthyosis-like skin lesions, Alagille syndrome or immune dysregulation. The information of four Chinese pediatric patients with *DCDC2*-related NSC in the Children's Hospital of Fudan University were reviewed. Clinical, pathological, and molecular features of all patients were analysed by clinical manifestations, laboratory investigations, liver biopsy, immunohistochemistry, and molecular genetic analysis. This study conformed to the provisions of the institutional ethics committee and the Declaration of Helsinki (as revised in 2013). The patients' parents shared all procedures including treatment and signed the written informed consent. The written informed consents were obtained for publication of any potentially identifiable images or data included in this paper.

### Liver biopsy

Archival formalin-fixed, paraffin-embedded liver biopsy samples from patient 1 and 2 were obtained from surgical biopsy. For each patient, tissue sections were cut at 4 µm and stained with haematoxylin-eosin (HE), and special staining for periodic acid–Schiff (PAS), masson, reticulin, copper, and iron.

### Immunohistochemistry

4-µm sections were deparaffinized, rehydrated, and pretreated with 3% H_2_O_2_ to eliminate endogenous peroxidase activity. Moreover, they were treated with EDTA (pH 9) or citrate buffer (pH 6) for heat-mediated antigen retrieval before commencing with the immunohistochemical staining protocol. The antibodies used included CK7, CK19, CD68, CD163, CD3, and CD8, which were purchased from http://www.maxim.com.cn (Fuzhou, China). The method of application is carried out according to the instruction manual. Finally, the sections were treated with DAB and counterstained with hematoxylin. In addition, a rabbit polyclonal antibody against *DCDC2* (purchased from http://www.ptgc.com, product code: 26978-1-AP) with a 1:200 dilution was utilized. The specific experimental procedures were carried out in accordance with the instructions. A normal liver sample (donated by a surgical patient) was prepared as the positive control, simultaneously omitting the first antibody as the negative control.

### Molecular genetic analysis

EDTA-anticoagulated whole blood specimens were used for the patients and their parents. DNA was extracted from peripheral blood. DNA libraries were prepared following the manufacturer's instructions, then sequenced on the Illumina platform. The whole exome capture high-throughput sequencing technology was utilized, with average coverage of 90-110X. Variants were detected using Genome Analysis Tool Kit (GATK) software (version: v3.2), including base quality score recalibration, InDels position and variant quality score recalibration, SNVs and InDels variant discovery and typing. And the AD:DP:GQ:PL was 13,5:18:61:61,0,420. The variants were annotated using Variant Effect Predictor (VEP) software, in which the functional coding regions and splicing site variants were called for further analysis, mainly including loss-of-function variants (mutations to obtain stop codons, frameshift mutations, and critical splicing point mutations), missense mutations, and non-frameshift deletions/insertions. Data were aligned against the Human Gene Mutation Database (HGMD) Professional (http://www.hgmd.cf.ac.uk), 1000 Genome Database (www.1000 genomes.org), Genome Aggregation Database (gnomAD) (https://gnomad.broadinstitute.org), dbSNP152 (https://www.ncbi.nlm.nih.gov/snp), and Exome Aggregation Consortium (ExAC) (http://exac.broadinstitute.org). Damage prediction of the genetic variants was conducted by Mutation Significance Cutoff (MSC) (https://lab.rockefeller.edu/casanova/MSC), which was applied to CADD (https://cadd.gs.washington.edu), PolyPhen 2 (http://genetics.bwh.harvard.edu/pph2) and SIFT (https://sift.bii.a-star.edu.sg/www/SIFT_indels2.html), with a confidence interval of 99% and database source of HGMD and ClinVar (https://www.ncbi.nlm.nih.gov/clinvar). The pathogenicity of amino acid changes caused by gene mutations was predicted by MutationTaster (http://www.mutationtaster.org) as well. According to the American College of Medical Genetics and Genomics (ACMG) guidelines, genetic variants were classified as pathogenic, likely pathogenic, variants of uncertain significance (VUS), likely benign, and benign. Thereafter, pathogenic variants of *DCDC2* were identified with the transcript of NM_001195610, which were validated by Sanger sequencing in patient 1. We conducted protein modeling by SWISS-model (https://www.swissmodel.expasy.org) with an UniProtKB code Q9UHG0, and the mutated structures were analyzed and visualized using PyMol (http://www.pymol.org).

### Literature search

In addition, we conducted a retrospective analysis of the literature. We used NSC and *DCDC2* as the terms, and retrieved a total of 7 related literatures from Pub Med, Spring Link, CNKI and Wan fang databases. There were 17 *DCDC2*-related NSC, summing to 21 cases including 4 cases in our paper. Then, we summarized and analyzed their clinical, pathological and molecular characteristics.

## Results

### Clinical features

There are four patients (2 female and 2 male) from 3 families, among whom patient 3 and 4 are siblings. All parents denied consanguinity. The age distribution is 6 months to 15 years. All patients presented with jaundice as the primary symptom in the first week of birth. Three patients (Patient 1, Patient 3 and Patient 4) suffered from recurrent respiratory infections during the course of the disease. Patient 1 developed sepsis and enteritis due to staphylococcus aureus and enterovirus infections during her first hospitalization. Patient 4 was infected with cytomegalovirus (CMV) and Epstein-Barr virus (EBV) after birth. Moreover, he developed into biliary cirrhosis at 10 months of age. Developmental delay and other medical history in four patients were unremarkable. Physical examination revealed the livers were located 3–5 cm below the right costal and the spleens 1–6 cm below the left costal. Spider nevus of the face in patient 4 was positive. Other physical examinations showed no positive signs. Biochemical findings included liver dysfunction with elevated total bile acid, total hyperbilirubinemia and GGT. Other blood tests except coagulation function showed no obvious abnormalities. Laparoscopic cholangiography was performed in patient 1 and 2, that revealed bile duct patency and dysplasia of the intrahepatic biliary tree. Magnetic resonance and cholangiopancreatography in patient 3 showed multiple and irregular dilatations of intrahepatic bile ducts. Hepatobiliary isotope imaging excluded biliary atresia (BA) (11). Computed tomography scan and angiography in patient 4 showed hydrocephalus and aneurysm of the communicating segment of the left internal carotid artery with vascular malformation (11). The clinical data and major lab investigations of four patients were listed in Table 1. All patients were treated with Ursodeoxycholic acid, Cholestriamine and took vitamin K1/A/E/AD supplements. The symptoms of all patients were relieved and the blood test indicators improved during regular examination.

**Table 1:** The clinical data and predominant laboratory investigations of four Chinese patients.

Patient	Patient1	Patient2	Patient 3	Patient 4
			Siblings	
Gender	Female	Female	Male	Male
Age	6 months	3 years	8 years	15 years
Onset age	2 days	5 days	3 days	3 days
Presenting symptoms	Jaundice	Jaundice	Jaundice	Jaundice
Developmental delay	None	None	None	None
Other manifestation	Sepsis	None	Recurrent respiratory tract infections	Recurrent respiratory tract infections
	Entericadenovirus infection			EBV infection
	Respiratory tract infections			CMV infection
Alanine aminotransferase (7–40 IU/L)	75.65	138.1	110	445.4
Aspartate aminotransferase (0–40 IU/L)	197.01	215.3	141	170.9
Alkaline phosphatase (54–369 IU/L)	508.48	675	811	557
*γ*-glutamyl transpeptidase (8-57 IU/L)	956.71	832	390	1042
Total bilirubin (5.1-17.1*μ*mol/L)	111.4	193.1	175.2	236.3
Direct bilirubin (0-6 μmol/L)	90.5	164.7	108.6	165.6
Total bile acid (0-10μmol/L)	321.5	91.6	304.4	NA
MRCP	NA	NA	Multiple cystic dilatations of intrahepatic bile ducts	NA
Laparoscopic cholangiography	Bile duct patency	The hilar bile duct was clouded and bile duct was unobstructed	Hepatobiliary isotope imaging excluded biliary atresia	NA

MRCP, magnetic resonance cholangiopancreatography; NA, not available.

### Liver biopsy

**Figure 1:**
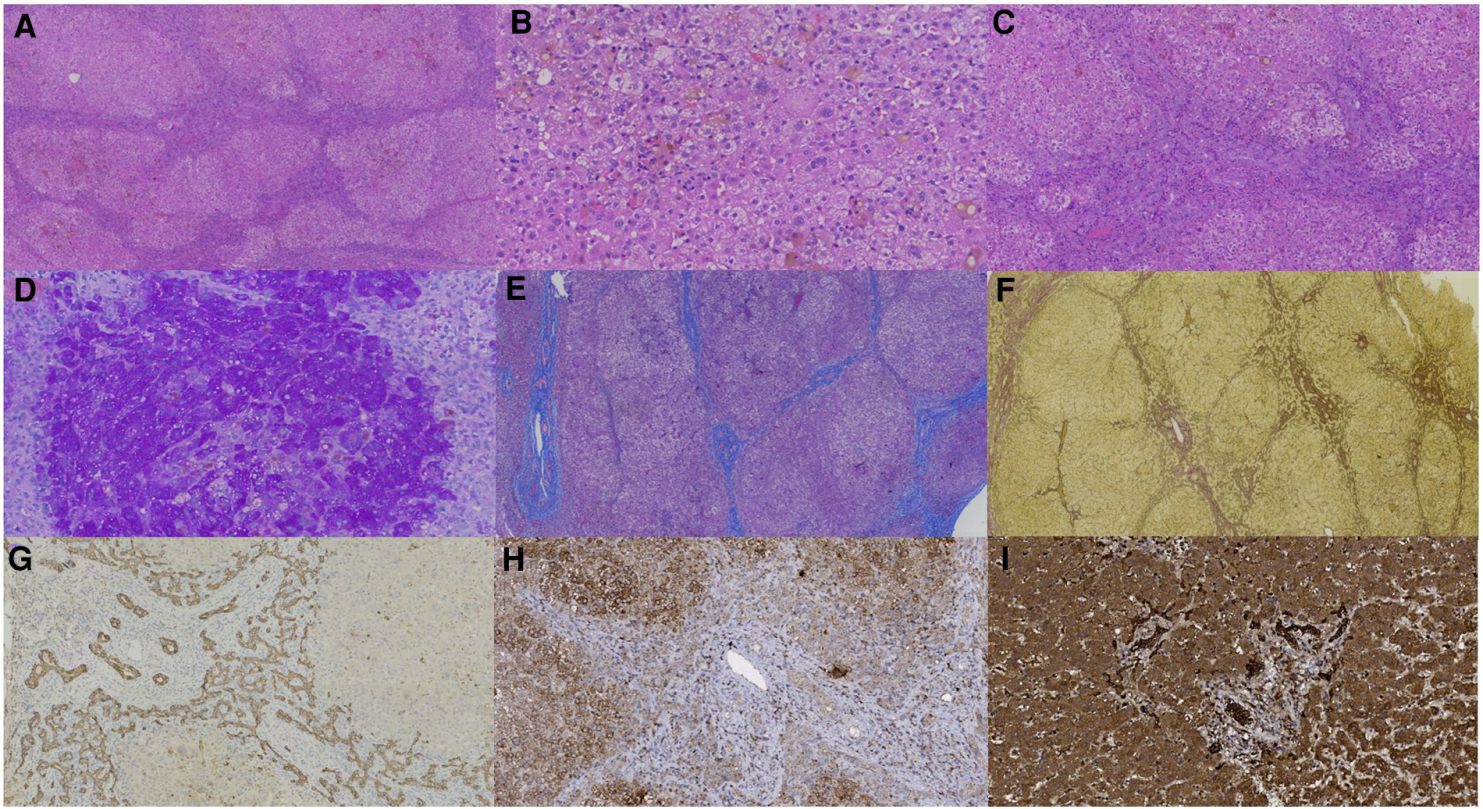
Liver biopsy (all originally magnified principal images). Hematoxylin and eosin staining showed the discorded architecture of liver lobules and the formation of pseudo-lobules (**A** × 40), balloon-like hepatic cells, giant cell changes of hepatocytes and bile plugs formation in hepatocytes and capillary bile ducts (**B** × 200), and ductular proliferation, moderate portal-tract inflammation, and fibrosis in the portal area (**C** × 100). PAS staining identified small vacuoles in partial hepatocytes (**D** × 200). Formation of bridging-like fibrosis and pseudo-lobule was highlighted by Masson staining (**E** × 50). The preserved reticular scaffold structure was displayed by reticulin staining (**F** × 40). Obvious hyperplasia of bile ducts along the margins of pseudo-lobule was showed by immunohistochemical staining of CK7 (**G** × 100). *DCDC2* immunohistochemical staining showed weaker expression in patient 1 (**H** × 200) than in normal control (**I** × 200).

Liver biopsy of patient 1 showed that the structural destruction of liver lobule and the formation of pseudo-lobule (Figure 1A). High magnification exhibited balloon-like degeneration of hepatocytes, giant cell changes of hepatocytes and bile plugs formation in hepatocytes and capillary bile ducts (Figure 1B). Ductular proliferation, the formation of bile thrombus in bile duct, moderate portal-tract inflammation, and fibrosis were found (Figure 1C). PAS staining revealed small vacuoles in some hepatocytes (Figure 1D), while copper staining was negative. Liver lobules were separated by the hyperplastic fibrous tissue in the portal area, formatting bridging-like fibrosis and pseudo-lobules, which was identified by Masson staining (Figure 1E). Reticulin staining indicated the preserved reticular scaffold structure (Figure 1F). Iron staining showed a little iron deposition. Liver histopathology presented cholestatic cirrhosis with stage 4 based on the Scheurer histopathologic scoring system (15). The pathological features of the liver biopsy in patient 2 were similar to those in patient 1, but without pseudo-lobule formation. The degree of inflammation and fibrous tissue hyperplasia in the portal area was less severe. Liver histopathology was assessed with stage 3.

### Immunohistochemistry

In patient 1, CK7 (Figure 1G) and CK19 staining showed obvious hyperplasia of bile ducts around pseudo-lobules. CD68 and CD163 staining manifested moderate proliferated Kupffer cells in hepatic sinusoids, and lymphocytes infiltrating in the portal area were indicated by CD3 and CD8 staining. It was important to notice that the expression of *DCDC2* was significantly reduced in patient sample (Figure 1H), compared with the normal liver sample (Figure 1I), which showed a cytoplasmic positive pattern in hepatic and bile duct cells. The results of immunohistochemical staining in patient 2 and patient 1 were resemble.

### Molecular genetic analysis

**Figure 2:**
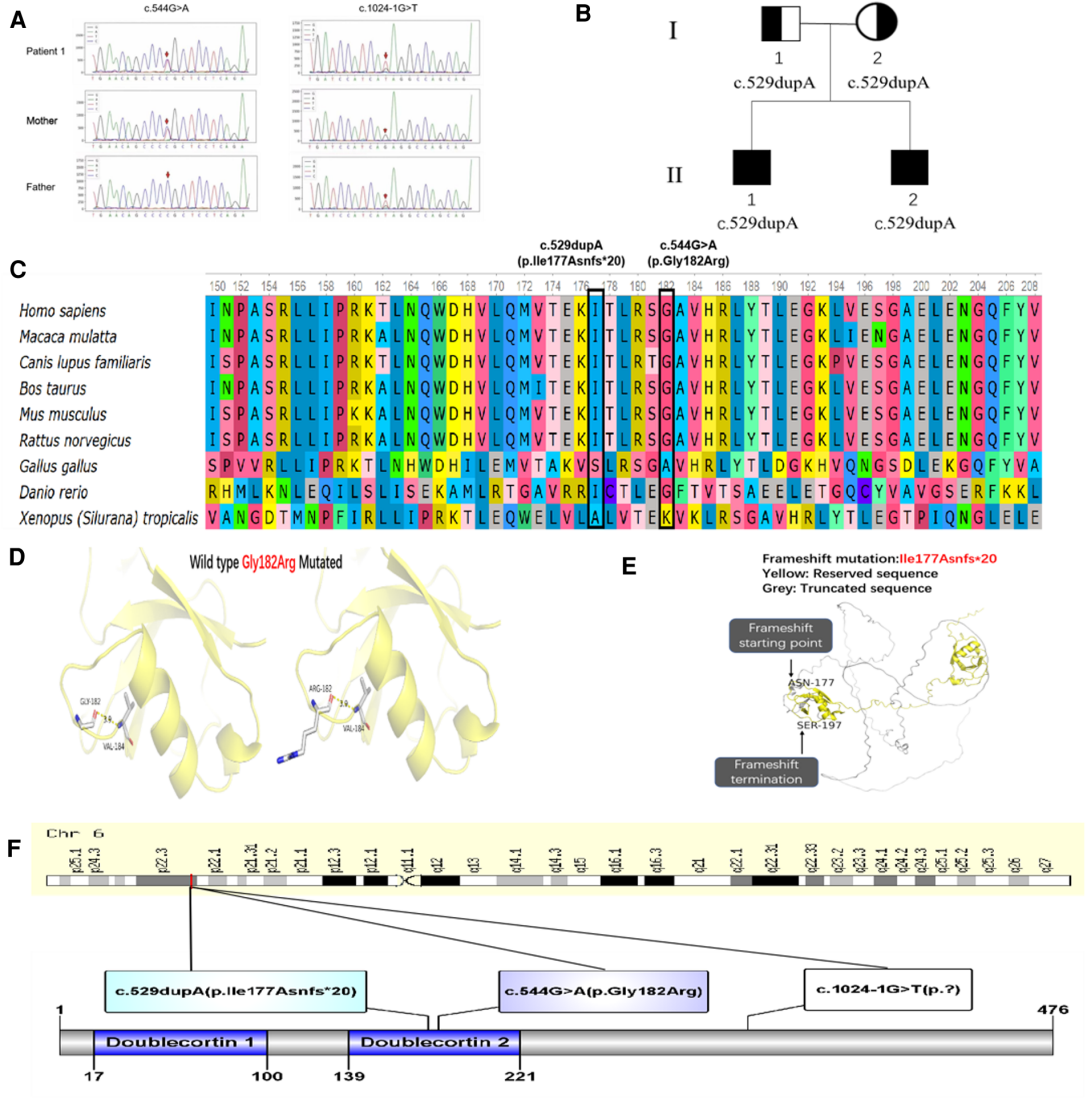
(**A**) Sanger sequencing confirmation of c.1024-1G > T and c.544G > A mutation of *DCDC2* in patient 1 and his parents. (**B**) Pedigree of patient 3 and 4, I-1 was the father, I-2 was the mother, II-1 was patient 3, II-2 was patient 4. (**C**) Conservation status of amino acid residues of the two variants across various species in four patients. (**D**) Wild and mutated types of the p. Gly182Arg variant in patient 1 compared by PyMol. (**E**) Spatial conformation of the frameshift mutation (p. Ile177Asnfs*20) showed by protein modeling. (**F**) Illustration of *DCDC2* protein domains, location of amino acid changes in four patients.

WES in 4 patients identified compound heterozygous variants c.1024-1G > T and c.544G > A (Figure 2A) in exon 5 and 10, and homozygous variant c.529dupA in exon 4 in *DCDC2*. In patient 1, The c.1024-1G > T was inherited from the healthy father and the c.544G > A was inherited from the healthy mother, resulting to the alteration of glycine to arginine at amino acid position 182 (p.Gly182Arg), which was conserved across various species (Figure 2C). In patient 3 and 4, the c.529dupA was inherited from their parents who were asymptomatic carriers (Figure 2B), which was reported in our publication. The MutationTaster score of c.1024-1G > T and c.544G > A was 1.0, both predicted as likely pathogenic by MSC. Both mutations of c.1024-1G > T and c.544G > A were assessed to be likely pathogenic in line with the guideline of the ACMG. The nomenclature of variants was on the basis of the recommendations of the Human Genome Variation Society (HGVS, http://www.hgvs.org/varnomen). Wild-type and mutated *DCDC2* were modeled by PyMol (http://www.pymol.org) which substantiated the change of Gly182Arg residue had no effect on the polar contact with them around amino acid Val 184 (Figure 2D). Spatial conformation of the frameshift mutation (p. Ile177Asnfs*20) predicted by protein modeling demonstrated that the mutated amino acid at position 177 moved backward 20 positions and then terminated, generating protein truncation (Figure 2E). Figure 2F illustrated *DCDC2* protein domains and the location of amino acid changes in our report.

### Retrospective analysis of the literature

The main features of twenty-one cases of *DCDC2*-related NSC were displayed in Table 2. The twenty-one patients with *DCDC2*-related NSC included sixteen Asian and Europeans (nine originating from Caucasian, Arabic and Turkish and seven from China). The male to female ratio approximates 3:1. The occurrence of NSC among siblings and high parental consanguinity implies autosomal recessive inheritance (16). Our study demonstrates forty-three percent of patients have parental consanguinity and fifty percent of patients are siblings. Most patients develop the disease within the first six months of life, with jaundice and/or acholic stools as the earliest symptoms. Upper gastrointestinal bleeding, ascites, hepatosplenomegaly and coagulopathy occurred in some patients at different stages of the disease. Biochemical test in all patients showed liver dysfunction with total hyperbilirubinemia and GGT with or without coagulation dysfunction. Five patients suffered from recurrent respiratory tract infections, one of whom had sepsis and enteric adenovirus infection and the other had CMV and EBV infection in our report, which may suggest impaired immune function in NSC patients. The renal involvement was found in four patients. Aneurysms were present in 2 patients (P4 and P9). Developmental delay and hypotonia were reported in 2 patients (P20 and P21). One patient (P6) had mild intellectual disability. These studies attest that *DCDC2*-related NSC mainly makes impact on the liver and meanwhile, urinary, nervous and muscular systems may be implicated. 13 individuals (62%) in 21 patients suffered from NSC received LT for end-stage liver disease at an average age of 11.9 years old (range 1–25). In all, three patients died among two of whom died of end-stage liver disease when awaiting for LT and one died after LT for complications of severe renal failure. Overall mortality was 14%.

**Table 2:** Main features of the twenty-one reported patients with NSC caused by mutations in *DCDC2.*

Patient	Mutation sites	Amino acid change	Exon	Sex	Country of origin	Parental consanguinity	Symptoms at onset	Age at onset	Age of LT	Other medical history	Liver histology	Reference
1	c.1024-1G > T/c.544G > A	p.?/p.Gly182Arg	10 and 5	Female	China	No	Jaundice	2 days	No LT	Sepsis, Enteric adenovirus infection, Recurrent respiratory tract infections	Pseudo-lobule formation, Ductular proliferation, bile plugs, portal-tract inflammation and fibrosis. Balloon-like hepatic cells and giant cell changes of hepatocytes. Biliary pattern cirrhosis.	This report
2	c.529dupA	p.Ile177AsnfsTer20	4	Female	China	No	Jaundice	5 days	No LT	None	Ductular proliferation, portal-tract inflammation and fibrosis. Balloon-like hepatic cells and giant cell changes of hepatocytes.	This report
3	c.529dupA	p.Ile177AsnfsTer20	4	Male	China	No	Jaundice	3 days	No LT	Recurrent respiratory tract infections	NA	This report
4 (Sibling of 3)	c.529dupA	p.Ile177AsnfsTer20	4	Male	China	No	Jaundice	3 days	No LT	Recurrent respiratory tract infections, EBV infection, CMV infection, Aneurysm	NA	This report
5	c.51G > C	p.Lys17Asn	1	Male	NR	Yes	Jaundice acholic stools	NR	25y	None	Early portal fibrosis, bile duct proliferation and biliary plugs. Cirrhosis.	Girard et al.
6 (Sibling of 5)	c.51G > C	p.Lys17Asn	1	Male	NR	Yes	Jaundice acholic stools	NR	14y	Unilateral ureteral duplication, Intellectual disability	Early portal fibrosis, bile duct proliferation and biliary plugs. Cirrhosis.	Girard et al.
7	c.426_557del	p.Phe142_Arg186del	4	Female	NR	Yes	Jaundice acholic stools	NR	6y	None	Early portal fibrosis, bile duct proliferation and biliary plugs. Cirrhosis.	Girard et al.
8 (Sibling of 7)	c.426_557del	p.Phe142_Arg186del	4	Male	NR	Yes	Jaundice acholic stools	NR	3.5y	None	Early portal fibrosis, bile duct proliferation and biliary plugs. Cirrhosis.	Girard et al.
9	c.649A > T	p.Lys217Ter	5	Female	Asian	Yes	Jaundice, Pale stools	20 weeks	No LT, Die	End-stage renal disease, Aneurysm	NR	Grammatikopoulos et al.
10	c.890 T > A	p.Leu297Ter	7	Female	Caucasian	No	Jaundice, Gastrointestinal bleeding, ascites	21 weeks	10y	None	Porto-portal bridging fibrosis. Ductular reaction with ductal bile plugs. Giant cell changes of hepatocytes. Biliary pattern cirrhosis	Grammatikopoulos et al.
11	c.757insG	p. Ser253ArgfsTer4	6	Male	Arabic	Yes	Jaundice, Gastrointestinal bleeding	6 weeks	14y	None	Ductal plate malformation. Ductal bile plugs. Hepatocellular cholestasis and giant cell change. Biliary pattern cirrhosis.	Grammatikopoulos et al.
12	c.529dupA/c.890 T > A	p. Ile177AsnfsTer20/ p.Leu297Ter	4 and 7	Female	Caucasian	No	Jaundice	4 weeks	15y, Die	None	Porto-portal bridging fibrosis and partial nodularity. Peripheral ductopaenia. Ectasia and cystic dilatation of perihilar bile ducts.	Grammatikopoulos et al.
13 (Sibling of 12)	c.529dupA/c.890 T > A	p. Ile177AsnfsTer20/ p.Leu297T	4 and 7	Female	Caucasian	No	Jaundice	1 weeks	14y	None	Ductal plate malformation. Ductal bile plugs. Giant cell changes of hepatocytes. Biliary pattern cirrhosis.	Grammatikopoulos et al.
14	c.123_124delGT/c.890 T > A	p. Ser42GlnfsTer72/ p.L297*	1 and 7	Male	Caucasian	No	Jaundice, Pale stools	6 weeks	No LT	None	Porto-portal bridging fibrosis. Ductular proliferation. Canalicular and hepatocellular cholestasis. Giant cell changes of hepatocytes.	Grammatikopoulos et al.
15	c.123_124delGT	p. Ser42GlnfsTer72	1	Male	Caucasian	No	Jaundice	7 weeks	15y	Hepatorenal syndrome	Porto-portal bridging fibrosis. Ductular proliferation and ductal bile plugs. Canalicular cholestasis. Giant cell change of hepatocytes. Biliary pattern cirrhosis.	Grammatikopoulos et al.
16	c.977_1001dup	p. Val335LeufsTer14	NR	Male	China	NR	jaundice	1 month	No LT, Die	None	Mild fibrosis of portal tracts.	Chen et al.
17	c.294-2A > G	p.?	NR	Male	NR	Yes	jaundice	After birth	1y	None	Micronodular cirrhosis. Chronic hepatocellular and canalicular cholestasis, with giant-cell change of hepatocytes, ductal-plate malformation.	Vogel et al.
18	c.705-2A > G/c.923_1023del	p.?	8	Male	China	NR	jaundice	3 days	23y	None	Early portal fibrosis and bile duct proliferation.	Lin et al
19 (Sibling of 18)	c.705-2A > G/c.923_1023del	p.?	8	Male	China	NR	jaundice	3 days	12y	None	NR	Lin et al
20	c.367_368del	p. Ser123GlnfsTer9	3	Male	Turkish	Yes	Jaundice	2 weeks	No LT	Recurrent respiratory tract infections, Nephronophthisis Developmental delay, Hypotonia	Bilirubin stasis, cholangiolytic changes, and septal fibrosis	Syryn et al
21	c.73G > A	p.Gly25Arg	NR	Male	Syria	Yes	Jaundice	3 days	2.8y	Recurrent respiratory tract infections, Developmental delay, Microcephaly, Hypotonia	Biliary-type cirrhosis	Syryn et al

NA, not available; NR, not reported; LT, liver transplant.

Liver biopsies were performed in seventeen patients, five of whom developed into biliary cirrhosis and others showed various degrees of liver fibrosis, including portal fibrosis, portal tract inflammation, bile duct proliferation or plate malformation with or without ductal bile plugs, giant cell changes of hepatocyte. It's worth noting that results of the histology on liver biopsies bring to mind the biliary atresia and congenital hepatic fibrosis, which makes it challenging to establish the diagnosis of NSC. *DCDC2* immunohistochemical staining was performed in some patients, which revealed *DCDC2* staining was heighten in the cytoplasm of cholangiocytes while a lesser extent or absent at the apical margin of cholangiocytes (4, 6, 10). These findings suggest that mutations in *DCDC2* perturbed protein expression and localization. The ultrastructural characteristics of hepatocytes were evaluated using a transmission electron microscope (TEM) in individual patient. In liver tissue, lobular cytoplasmic necrosis, dilatation of canalicular lumina with amorphous bile, were seen as well as blunting of microvilli, and cytoplasmic blebbing into the canalicular lumen, while cholangiocellular primary cilia were existent or absent (6, 10).

## Discussion

NSC is a rare hereditary liver disease in nurseling, for which *DCDC2* mutation was mainly responsible (4, 6). With the development of WES, researchers found biallelic mutations in *DCDC2* can cause familial NSC (13). The *DCDC2* is located on chromosome 6p22.3, containing 11 exons, which encodes *DCDC2* protein known to bind tubulin and enhance microtubule polymerization (17). Grati et al. found the pathogenicity of *DCDC2* mutations in the aspects of protein expression and localization as well as their influence on ciliogenesis in cholangiocytes (7). The primary cilia of cholangiocytes are important for the regulation of bile flow and its composition (18). Grammatikopoulos et al. suggested that the absence of *DCDC2* may be related to the formation of “cytotoxic” bile or the dysregulation of the cholangiocyte's homeostatic mechanisms, possibly by Wnt signalling pathway (8). *DCDC2* has been shown to interact with KIF3A, a subunit of the Kinesin-2 complex that is essential for cilia formation and maintenance, which can modulate ciliary signaling by Wnt signaling pathway (17). *DCDC2* negatively regulates Wnt/*β*-catenin signaling pathway by interacting with dishevelled 3, a key regulator of the pathway (17). The deletion of the second doublecortin domain of *DCDC2* also has been shown to hold back the decrease of Wnt/*β*-catenin signaling in the case of Wnt inhibitors (8). Therefore, the inhibitor of Wnt signaling pathway perhaps be a promising treatment for patients with NSC to prevent the progression of the disease.

It was reflected in our report that NSC are more common among siblings and parental consanguinity. The symptoms of the four Chinese patients at onset we reported were consistent with those documented in the literatures. Three patients had the history of upper respiratory infection, one of whom had aneurysm. Two of our patients had a history of virus infection, including enteric adenovirus, EBV, and CMV. Developmental delay, hypotonia, intellectual disability microcephaly and unilateral ureteral duplication reported in the literatures (7–10) were not found in our patients. At present, it is not very clear whether nerve, muscle, or immune system involvements are related to mutations in *DCDC2*. Researchers hypothesized that the involvement of these systems may be related to the inactivation of the cilium function or the disorder of Wnt signaling pathways due to *DCDC2* mutations (10). More cases and further studies are needed to confirm this hypothesis. None of our four patients either received a liver transplant or died, including one with severe cirrhosis. Our patients were given Ursodeoxycholic acid capsules, Cholestyramine, Vitamins A, D, E and K1. Liver function and symptoms in all patients have improved. However, two patients have the early cirrhosis, which makes the outcome of the disease disappointed. The final outcome still needs further follow-up.

In terms of liver biopsy pathology, the pathological changes of our patients were similar to those with *DCDC2*-related NSC reported in the literature, showing bile duct proliferation, fibrosis and ductal bile plugs at different degrees with or without giant cell changes of hepatocytes. The histological changes of liver biopsy were similar to those reported in the previous literature.

We also reported a novel mutation (c.1024-1G > T /p.? and c.544G > A /p. Gly182Arg) in *DCDC2*. What's more, *DCDC2* immunohistochemical staining revealed that the expression level of *DCDC2* protein in NSC patients was significantly decreased, compared with normal liver tissue. It demonstrated that *DCDC2* mutation affected protein expression, which was consistent with published literatures. But its effect on protein function needs to be determined in future study. Besides, we sum up the amounts of patients suffering from NSC with mutations in *DCDC2* to 21 from 16 irrelevant families and pathogenic variants to 15. These variants include 4 non-truncating, 8 truncating, and 3 splice site variants (Table 2), which will give a more complete picture of *DCDC2*-related NSC.

In this paper, we discovered novel mutations in *DCDC2* causing NSC in 4 Chinese children, which extended the genetic spectrum of *DCDC2*. For the moment, there is no specific treatment for patients with NSC, and patients with biliary cirrhosis require liver transplantation for survival.

## Data Availability

The data presented in this study is included in the article/Supplementary Material. The DNA datasets are not readily available due to privacy restrictions, further inquiries should be directed to the corresponding authors.

## References

[1] Chen HL Wu SH Hsu SH Liou BY Chen HL Chang MH. Jaundice revisited: recent advances in the diagnosis and treatment of inherited cholestatic liver diseases. J Biomed Sci. (2018) 25(1):75. 10.1186/s12929-018-0475-8 30367658 PMC6203212

[2] Amedee-Manesme O Bernard O Brunelle F Hadchouel M Polonovski C Baudon JJ Sclerosing cholangitis with neonatal onset. J Pediatr. (1987) 111(2):225–9. 10.1016/s0022-3476(87)80072-0 3612394

[3] Kerkar N Chan A. Autoimmune hepatitis, sclerosing cholangitis, and autoimmune sclerosing cholangitis or overlap syndrome. Clin Liver Dis. (2018) 22(4):689–702. 10.1016/j.cld.2018.06.005 30266157

[4] Girard M Bizet AA Lachaux A Gonzales E Filhol E Collardeau-Frachon S DCDC2 Mutations cause neonatal sclerosing cholangitis. Hum Mutat. (2016) 37(10):1025–9. 10.1002/humu.23031 27319779

[5] Hadj-Rabia S Baala L Vabres P Hamel-Teillac D Jacquemin E Fabre M Claudin-1 gene mutations in neonatal sclerosing cholangitis associated with ichthyosis: a tight junction disease. Gastroenterology. (2004) 127(5):1386–90. 10.1053/j.gastro.2004.07.022 15521008

[6] Grammatikopoulos T Sambrotta M Strautnieks S Foskett P Knisely AS Wagner B Mutations in DCDC2 (doublecortin domain containing protein 2) in neonatal sclerosing cholangitis. J Hepatol. (2016) 65(6):1179–87. 10.1016/j.jhep.2016.07.017 27469900 PMC5116266

[7] Grati M Chakchouk I Ma Q Bensaid M Desmidt A Turki N A missense mutation in DCDC2 causes human recessive deafness DFNB66, likely by interfering with sensory hair cell and supporting cell cilia length regulation. Hum Mol Genet. (2015) 24(9):2482–91. 10.1093/hmg/ddv009 25601850 PMC4383862

[8] Schueler M Braun DA Chandrasekar G Gee HY Klasson TD Halbritter J DCDC2 Mutations cause a renal-hepatic ciliopathy by disrupting wnt signaling. Am J Hum Genet. (2015) 96(1):81–92. 10.1016/j.ajhg.2014.12.002 25557784 PMC4289677

[9] Meng H Smith SD Hager K Held M Liu J Olson RK DCDC2 Is associated with Reading disability and modulates neuronal development in the brain. Proc Natl Acad Sci USA. (2005) 102(47):17053–8. 10.1073/pnas.0508591102 16278297 PMC1278934

[10] Syryn H Hoorens A Grammatikopoulos T Deheragoda M Symoens S Vande Velde S Two cases of DCDC2-related neonatal sclerosing cholangitis with developmental delay and literature review. Clin Genet. (2021) 100(4):447–52. 10.1111/cge.14012 34155636

[11] Li JQ Lu Y Qiu YL Wang JS. Neonatal sclerosing cholangitis caused by DCDC2 variations in two siblings and literature review. Zhonghua Er Ke Za Zhi. (2018) 56(8):623–7. Chinese. 10.3760/cma.j.issn.0578-1310.2018.08.013 30078246

[12] Vogel GF Maurer E Entenmann A Straub S Knisely AS Janecke AR Co-existence of ABCB11 and DCDC2 disease: infantile cholestasis requires both next generation sequencing and clinical-histopathologic correlation. Eur J Hum Genet. (2020) 28(6):840–4. 10.1038/s41431-020-0613-0 32203204 PMC7253416

[13] Lin Y Zhang J Li X Zheng D Yu X Liu Y Biallelic mutations in DCDC2 cause neonatal sclerosing cholangitis in a Chinese family. Clin Res Hepatol Gastroenterol. (2020) 44(5):e103–8. 10.1016/j.clinre.2020.02.015 32205117

[14] Chen J Zhang XX Liu HD Chen X. Neonatal sclerosing cholangitis caused by a novel DCDC2 gene variant: a case report and literature review. Chin Pediatr Emerg Med. (2020) 27(2):158–60. 10.3760/cma.j.issn.1673-4912.2020.02.019

[15] Scheuer PJ. Classification of chronic viral hepatitis: a need for reassessment. J Hepatol. (1991) 13(3):372–4. 10.1016/0168-8278(91)90084-o 1808228

[16] Debray D Pariente D Urvoas E Hadchouel M Bernard O. Sclerosing cholangitis in children. J Pediatr. (1994) 124(1):49–56. 10.1016/s0022-3476(94)70253-5 8283375

[17] Massinen S Hokkanen ME Matsson H Tammimies K Tapia-Páez I Dahlström-Heuser V Increased expression of the dyslexia candidate gene DCDC2 affects length and signaling of primary cilia in neurons. PLoS One. (2011) 6(6):e20580. 10.1371/journal.pone.0020580 21698230 PMC3116825

[18] Banales JM Huebert RC Karlsen T Strazzabosco M LaRusso NF Gores GJ. Cholangiocyte pathobiology. Nat Rev Gastroenterol Hepatol. (2019) 16(5):269–81. 10.1038/s41575-019-0125-y 30850822 PMC6563606

